# Novel 3D Hybrid Nanofiber Scaffolds for Bone Regeneration

**DOI:** 10.3390/polym12030544

**Published:** 2020-03-02

**Authors:** Dorota Kołbuk, Marcin Heljak, Emilia Choińska, Olga Urbanek

**Affiliations:** 1 Institute of Fundamental Technological Research Polish Academy of Sciences, Adolfa Pawińskiego 5b, 02-106 Warsaw, Poland; ourbanek@ippt.pan.pl; 2 Faculty of Materials Sciences and Engineering, Warsaw University of Technology, Woloska 141, 02-507 Warsaw, Poland; marcin.heljak@pw.edu.pl (M.H.); choinska.emilia@gmail.com (E.C.)

**Keywords:** hybrid scaffolds, electrospinning, freeze-drying, gelatin, hydroxyapatite, sonochemical covering/grafting

## Abstract

Development of hybrid scaffolds and their formation methods occupies an important place in tissue engineering. In this paper, a novel method of 3D hybrid scaffold formation is presented as well as an explanation of the differences in scaffold properties, which were a consequence of different crosslinking mechanisms. Scaffolds were formed from 3D freeze-dried gelatin and electrospun poly(lactide-*co*-glicolide) (PLGA) fibers in a ratio of 1:1 *w*/*w*. In order to enhance osteoblast proliferation, the fibers were coated with hydroxyapatite nanoparticles (HAp) using sonochemical processing. All scaffolds were crosslinked using an EDC/NHS solution. The scaffolds’ morphology was imaged using scanning electron microscopy (SEM). The chemical composition of the scaffolds was analyzed using several methods. Water absorption and mass loss investigations proved a higher crosslinking degree of the hybrid scaffolds than a pure gelatin scaffold, caused by additional interactions between gelatin, PLGA, and HAp. Additionally, mechanical properties of the 3D hybrid scaffolds were higher than traditional hydrogels. In vitro studies revealed that fibroblasts and osteoblasts proliferated and migrated well on the 3D hybrid scaffolds, and also penetrated their structure during the seven days of the experiment.

## 1. Introduction

Various scaffold formation methods have been developed for tissue regeneration applications. It is crucial to be aware of their advantages and limitations as well as the influence of processing conditions on the (sub)molecular structure and properties of materials. Polymeric scaffolds may be formed using electrospinning [1,2], 3D printing [3,4,5], thermally induced phase separation (TIPS) [6], foaming [7] or leaching techniques [8,9], etc. However, all of these methods have disadvantages described in detail by others [10,11]. For example, the morphology of electrospun fibers mimics collagen fibers present in the extracellular matrix of the tissues. However, tight pores of electrospun nonwoven fibers do not enable effective scaffold penetration by the cells. A variety of approaches have been investigated to enhance the pore size of the electrospun nonwovens [12]. Alternatively, 3D printed scaffolds indicate a precisely designed pore size and architecture, although 3D printer resolution is limited and total porosity is less than 80% [13,14]. Moreover, the size of the filaments is large, compared to the cell dimensions [15]. Another form of currently investigated materials are hydrogels. For soft tissue regeneration, this form of material is beneficial due to its hydration. However, poor mechanical properties limit their use for load bearing applications. These reasons have contributed to the development of hybrid scaffolds. A hybrid scaffold combines the advantages of selected techniques such as electrospun fibers and hydrogels.

Besides the choice of scaffold fabrication method, a proper composition is essential for scaffold effectiveness. Collagen is one of the most common choices. It is the most abundant fibrous protein within the interstitial extracellular matrix (ECM) and constitutes up to 30% of the total protein mass of mammals [16,17]. Gelatin is a denatured form of the collagen; it is biocompatible and indicates specific amino acid sequences that interact with cells. Cells attach to the gelatin surface due to arginine–glycine–aspartate (RGD) sequences and amphoteric polyelectrolyte character of gelatin [18]. The amphoteric polyelectrolyte character of gelatin is related to its polarity. Inside the gelatin globule apolar groups occur, while polar and charged groups are often found outside the protein surface [19]. Strong, long-ranged electrostatic attraction between a charged adsorbent and oppositely charged amino acid side chains will lead to a significant free energy change favoring the adsorption process. The stability of such structure depends on the combination of hydrophobic interactions between the hydrophobic side chains: hydrogen bonds between the neighboring side chains and along the polypeptide chains, the Coulomb interactions between charged residues, and van der Waals interactions. Gelatin properties may be tailored by various factors such as temperature, pH, or type of solvent, because gelatin shows a large diversity of higher order molecular structures, being dependent on external conditions [20]. Two component scaffolds formed from blends of proteins, and polyesters are formed to reduce their weak elasticity [21].

Hybrid scaffolds are the answer to the deficiencies of scaffolds formed from material blends. Electrospun nonwovens impregnated with gel were developed for soft and hard tissue regeneration. Several methods of incorporating electrospun nonwovens and single fibers into hydrogel have been analyzed. Nonwovens incorporated into 3D hydrogels via a bottom-up, layer-by-layer assembly process were analyzed as a scaffold for nucleus pulposus regeneration [22] and bone tissue regeneration [23]. This idea is part of a biomimetic approach and led to the imitation of fibrous structures occurring in biological structures such as nucleus pulposus and osteon, respectively. Hybrid scaffolds made of rolled electrospun nonwoven fixed with a fibrin gel were prepared by McMahon et al. [24]. Electrospun nonwovens in the form of separated fibers were incorporated by several authors and tested as injectable systems for spinal cord healing [24] or cartilage regeneration [25,26]. Nonwovens were cut into small pieces and disintegrated on single fibers under sonification conditions [27], aminolysis [28], or UV [29]. As a complement to this topic, the review of Bosworth et al. is highly recommended. The review describes significant developments in the design of hybrid scaffolds composed of fibers and hydrogels [30].

Hybrid scaffolds fabricated using the layer by layer method or fabricated on the base of twisted electrospun nonwovens overcome the limitations of a single nonwoven sheet. They indicate a three-dimensional architecture and exhibit better mechanical properties. The pore size of electrospun nonwovens increases with the fiber diameter, but even for fibers with a mean diameter up to 1 µm, the pore size does not excide 20 µm [31,32]. Such pore sizes are still not enough for cellular migration [12].

All hybrid scaffolds composed of peptides have to be stabilized in order to slow down dilution processes under in vitro conditions. Several types of crosslinking factors may be used, both chemical (aldehydes, isocyanates, carbodiimides, etc.) and physical (ultraviolet irradiation, dehydrothermal treatments, etc.). EDC (1-ethyl-3-(3-dimethylaminopropyl-carbodiimide hydrochloride) in the presence of NHS (N-hydroxy-succinimide) is successfully used as a collagen and gelatin chemical crosslinker. Peptide bonds between primary amino groups in lysine residues and carboxyl groups on glutamate or aspartate residues are crosslinked. In the case of hybrid scaffolds composed of gelatin combined with nanoparticles such as hydroxyapatite (HAp), additional chemical bonds are formed due to the presence of Ca^2+^ ions.

The purpose of this work was to develop a new method of 3D hybrid scaffold formation, combining advantages of electrospun fibers and hydrogels as well as various materials. We developed a hybrid scaffold composed of gelatin in the form of a freeze-dried sponge, and electrospun fibers covered with hydroxyapatite for application in bone and teeth regeneration. The method of fibers covered with HAp has previously been developed [33] and was additionally modified for the purposes of this publication. Nonwoven porosity was significantly increased due to ultrasonic treatment; separated fibers were homogenously embedded in the hydrogel, so the cellular migration was not limited. The influence of the chemical composition of the scaffold and EDC/NHS crosslinking process on mechanical properties, water absorption, and cellular proliferation was evaluated.

## 2. Materials and Methods

### 2.1. Materials

Electrospun nanofibers were formed from poly(lactide-*co*-glicolode) (PLGA) (Resomer 855S, Evonik) with PLA:PGA in the ratio of 85:15. Hydrogel was prepared from gelatin (gelatin from porcine skin Type A, Sigma-Aldrich, St. Louis, MA, USA). As a solvent for PLGA, 1,1,1,3,3,3-hexafluoro-2-propanol (HFIP) (Iris Biotech GmBH, Marktredwitz, Germany) was used. Fibers were coated with hydroxyapatite nanoparticles (HAp) (nanoXIM-HAp 202, Fluidinova). For gelatin crosslinking *N*-hydroxysuccinimide (98%, Sigma Aldrich) and *N*-(3-Dimethylaminopropyl)-*N*′-ethylcarbodiimide hydrochloride (99%, Sigma Aldrich) were used.

### 2.2. Nonwoven Formation

PLGA was dissolved in HFIP at a concentration of 3% *w*/*w*. Nonwovens were electrospun with an applied voltage of +15 kV and a flow rate of 3 mL/h. The needle-collector distance was set at 18 cm. Drum rotation was 200 rpm and did not cause any orientation of the fibers. The electrospinning was conducted at room temperature and at a humidity of 40% ± 5%.

### 2.3. Hybrid Scaffold Preparation

**Figure 1:**
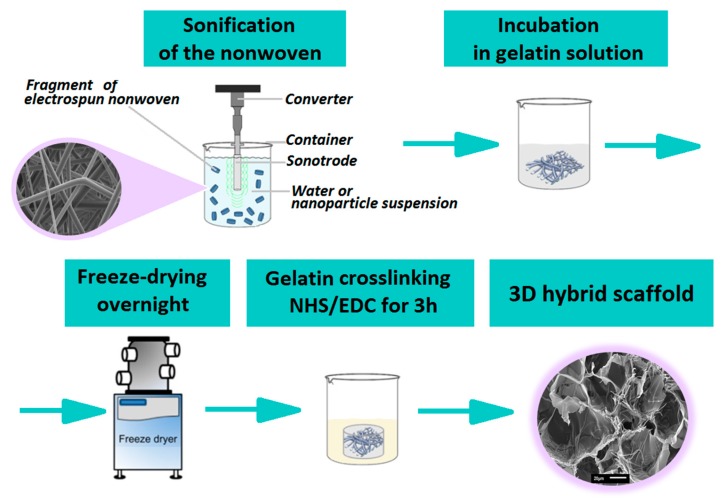
Scheme of the scaffold preparation.

The scheme of the scaffold preparation is illustrated in Figure 1. In the first step, ca. 5 mg pieces of nonwovens were cut and suspended in water. Such prepared fibers were subjected to ultrasound for 5 min with a total energy of 10,000 Ws and amplitude of 70% in pulse mode (Ultrasonic Homogenizer UP200Ht, Hielscher). The process was conducted in an ice bath in order to avoid fiber degradation at high temperature. After a sonochemical procedure, the tightly packed nonwoven structure was destroyed. The final form of the nonwoven suspended in water after this procedure was similar to a piece of cotton. Sonochemical covering of fibers with HAp was conducted as described by Kołbuk et al. [33] on previously sliced nonwovens.

After sonification, nonwoven pieces in the form of cotton, covered and non-covered with HAp, were placed in a 5% gelatin solution and kept for 1 h at 37 °C on a horizontal shaker. After this, the samples were placed in a 48-multiwall plate. Pure gelatin solutions without fibers were placed in the same way. Finally, all samples were frozen and freeze-dried overnight. The ratio of gelatin to fiber mass was 1:1 *w*/*w*. Then, all samples were crosslinked using EDC/NHS (1-ethyl-3-(3-dimethylaminopropyl)/carbodiimideN-hydroxysuccinimide) dissolved in 80% *v*/*v* ethanol for 3 h. The EDC/NHS crosslinking solution was prepared as described by Lai et al. [34]. After this process, samples were washed three times with an 80% ethanol solution and finally left for free evaporation and dried overnight in a vacuum dryer.

Scaffolds were named Ge (crosslinked freeze-dried gelatin without fibers), GeN (scaffold made of crosslinked freeze-dried gelatin and PLGA fibers), and GeNHap (scaffold made of crosslinked freeze-dried gelatin and PLGA fibers covered with HAp). As a reference in the selected experiments, some other samples were used: gelatin powder (Ge_powder), non-crosslinked freeze-dried gelatin (Ge_NC), PLGA fibers (NanoF), and HAp powder (HAp_powder).

### 2.4. Characterization

Imaging of the samples was conducted using scanning electron microscopy (SEM) (Jeol JSM-6010PLUS/LV InTouchScope™) at an accelerated voltage of 7 kV. Before imaging, the nonwovens were coated with gold.

Nanoparticles attached to the fibers’ surface were visualized using a transmission electron microscope (TEM) (Jeol JEM-1011). The GeNHap samples were embedded in epoxy resin and cut on an ultramicrotome. A TEM analysis was conducted using an accelerated voltage of 80 kV.

In order to reveal and prove chemical differences between the samples, Fourier transform infrared spectroscopy (FTIR, Vertex70 spectrophotometer, Bruker) was conducted. For each sample, 32 scans in the range of 400–4000 cm^−1^ were done with a resolution of 2 cm^−1^.

Hydroxyapatite presence was confirmed using wide angle x-ray diffraction (WAXS) (D8 Discover, Bruker). Measurements were done in reflection mode, using Bragg–Brentano geometry. The angular range of analyses (2Theta) was between 5–35 degrees, with a step of 0.01 degrees, and a time of data accumulation at a particular angular point of 0.2 s.

To determine hydroxyapatite content in the GeNHAp samples, thermogravimetric analysis (TGA) were performed (Q5000, TA Instruments). The analysis was done in a nitrogen atmosphere at a temperature range of 40–700 °C, with a heating rate of 10 °C/min. All samples were analyzed and their mass was ca.10 mg.

The mechanical behavior of the 3D hybrid scaffolds was investigated with the aim of (i) quantifying the compressive properties of the specimens through monotonic compression tests and (ii) measuring the viscoelastic properties by means of dynamic mechanical analysis (DMA) (Q800, TA Instruments). A DMA instrument equipped with compression clamps enabled it for testing in submersion. During the tests, all the samples were submerged in demineralized water [35,36]. In the case of the monotonic test, all the samples were preloaded to 0.01 N and compressed at a constant strain rate of 6%/minute. The scaffolds’ compressive moduli were identified as the ratio between compressive stress and strain in the linear portion of the curve (at 20% of total strain). To obtain the viscoelastic properties of the hybrid scaffolds, they were initially preloaded to 0.2 N and subjected to cyclic sinusoidal loading characterized by a displacement amplitude of 15 µm over a frequency range from 1 to 10 Hz [3]. The data obtained were used to calculate the storage *E*’ and the loss *E*” moduli as well as the tangent of the phase lag angle *δ* between stress and strain.

Water absorption tests were carried out at selected time points within three hours of incubation in distillated water at room temperature. Before the test, samples were dried in a vacuum dryer overnight. After each time of incubation, samples were slightly dried on a paper towel and weighed.

Cellular response on the surface of the 3D scaffolds and cell infiltration of their structure was analyzed using mice fibroblast cells (L929, Sigma-Aldrich ATTC) and human osteoblast MG63 (Sigma-Aldrich ATTC). Cells were cultured in a Dulbecco’s Modified Eagle Medium medium with 10% fetal bovine serum, 1% antibiotic, and kept in an incubator. An additional component in the medium for the MG63 cells was 1% glutamine. Before cell seeding, materials were sterilized in 80% ethanol and by UV radiation (30 min on each side of the sample). Cells were incubated with the analyzed samples for seven days in the incubator (37 °C, CO_2_).

Cellular viability was analyzed after one, three, and seven days of cell culture using a Presto Blue assay (Presto Blue, Invintrogen^TM^), according to the protocol. The incubation time was 1 h. The data were collected using fluorescence, with excitation at 530 nm and emission at 620 nm (Fluoroskan Ascent FL, Thermo Fisher Scientific). Additionally, scanning electron microscopy (JEOL JSM-6390LV) was used to image cell adhesion, morphology, and spreading on the scaffold. For SEM imaging, cells were fixed with 2.5% glutaraldehyde solution per 0.5 h and dehydrated with ethanol solutions, and finally dehydrated with ethanol/hexamethyldisiloxane. Finally, samples were coated with gold.

For florescent microscopy, samples were fixed with 3% paraformaldehyde solutionsolution for 30 min. Samples were stained with ActinGreen™ and NucBlue™ Reagent (Invintrogen^TM^). The samples were observed using fluorescent microscope (Leica DMI3000B, Leica Microsystems).

### 2.5. Statistical Analysis

Quantitative data of water absorption, mass loss, mechanical properties, and cytotoxicity were expressed as mean ± SD. Data were compared using a one-way ANOVA test and analysis of variance (Tukey’s test). The data were considered as significantly different when *p* < 0.05.

## 3. Results

**Figure 2:**
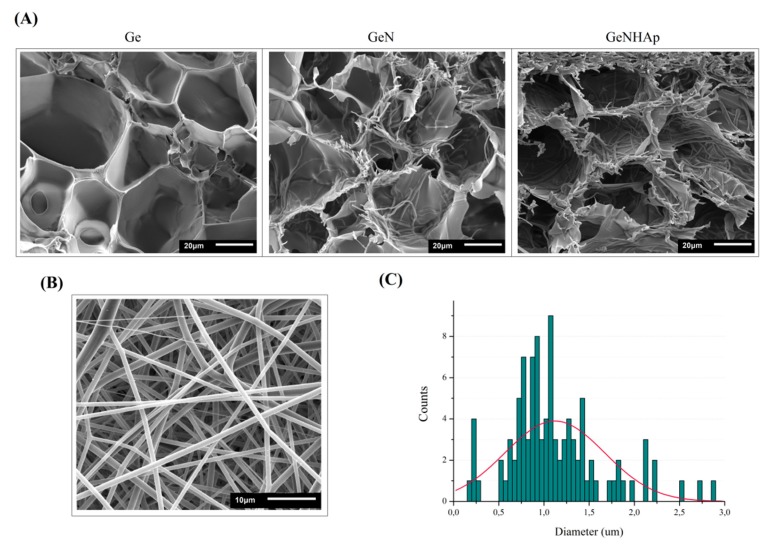
Morphology evaluation. (**A**) SEM images of the samples: Ge, GeN, GeNHAp; (**B**) SEM image of the PLGA fibers; (**C**) distribution of the fibers’ diameter.

**Figure 3:**
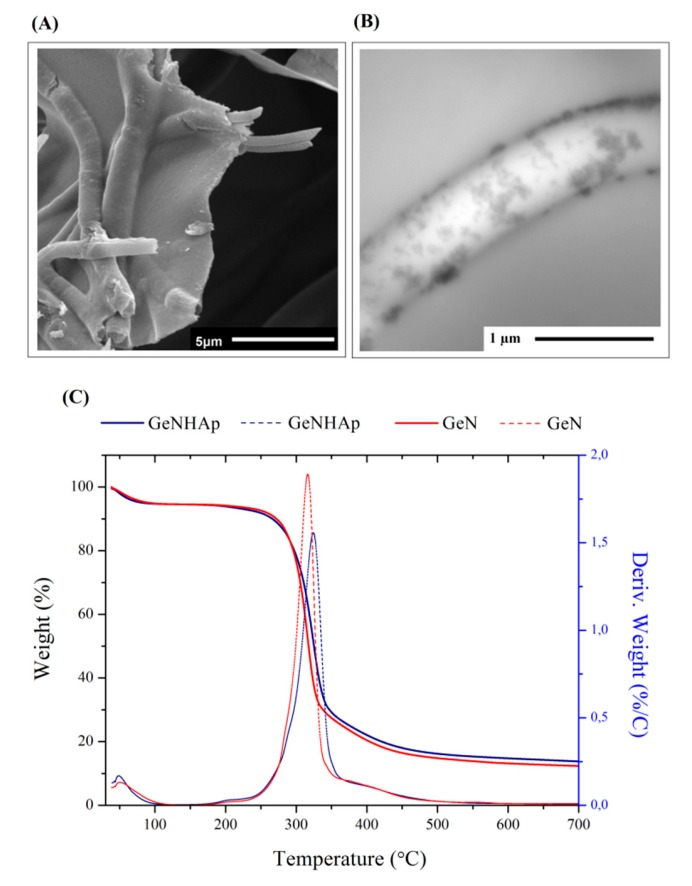
Analysis of fibers covered with HAp (GeNHAp). (**A**) SEM image presenting PLGA fibers embedded in the walls of freeze-dried Ge; (**B**) TEM image of a single fiber covered with HAp and embedded in freeze-dried gelatin solution; (**C**) thermogravimetric curves of the GeN and GeNHap samples. The data show the differences in the thermal behavior of each type of sample and determine the HAp amount in the GeNHAp sample.

Hybrid scaffolds were prepared by incorporation of electrospun fibers into a gelatin solution and ultimately freeze-dried. The SEM images illustrate the samples’ architecture: freeze-dried gelatin (Ge), gelatin with PLGA fibers (GeN), and gelatin with PLGA fibers covered with HAp (GeNHAp) (Figure 2A). The images confirm the high porosity of all scaffolds and uniform fiber distribution. Fibers were incorporated into gelatin in the form of disintegrated nonwovens (“cotton’’) prepared under sonochemical conditions (Figure 2B). These fibers were free of defects; the diameter of a single fiber was determined in a range of 0.2–2.8 µm, with average diameter of 1.12 µm, as shown in Figure 2C. Fibers in GeN and GeNHAp were nicely embedded in the pore walls of gelatin (Figure 3A) and contribute significantly to the increase of the surface variability of GeN and GeNHAp in comparison to Ge. PLGA fibers covered with HAp under sonochemical conditions were illustrated by TEM (Figure 3B). The TEM image confirms the presence of HAp on the fibers’ surface and a uniform distribution (Figure 3B). The amount of HAp in the GeNHAp scaffold was determined using a TGA analysis and it was calculated as c.a. 3% (Figure 3C).

**Figure 4:**
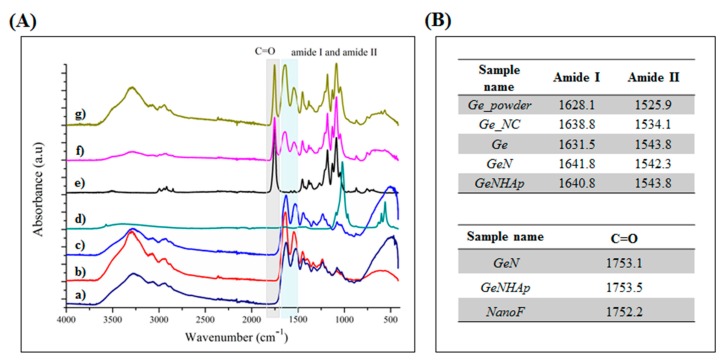
FTIR analysis. (**A**) Spectra of Ge_powder; (b) Ge_NC; (c) Ge; (d) HAp_powder; (e) NanoF; (f) GeN; (g) GeNHAp. (**B**) Tables-presenting wavenumbers in which spectra for selected chemical bonds occur.

The FTIR spectra of Ge, GeN, and GeNHAp in comparison to the non-crosslinked freeze-dried gelatin (Ge_NC), pure PLGA fibers (NanoF), HAp powder (HAp_powder), and Ge powder (Ge_powder) are presented in Figure 4. Gelatin indicates the four main signals related to amides: amide A (3290–3300 cm^−1^), amide I (1600–1700 cm^−1^), amide II (1510–1580 cm^−1^), and amide III (1230–1240 cm^−1^) [^i^]. PLGA indicated signals coming from various chemical bonds such as the C=O bond from both lactide and glicolide components (1756 cm^−1^), C–H (1360–1455 cm^−1^), C–O from the carboxyl group (1212 cm^−1^), C–O (1130 cm^−1^, 1185 cm^−1^), and C–CH_3_ (1046 and 1093 cm^−1^) [37]. A signal between 900–1150 cm^−1^ is characteristic for PO_3_^−4^ in HAp [33]. The 3D scaffolds GeN and GeNHAp indicated a combination of signals from appropriate components (Figure 4). Samples with nanofibers (GeN and GeNHAp) indicated a shift of amide I and amide II to lower values in comparison to Ge. Peaks corresponding with the C=O coming from lactide and glicolide components, present in GeN and GeNHAp, indicated a higher wavelength than in NanoF.

**Figure 5:**
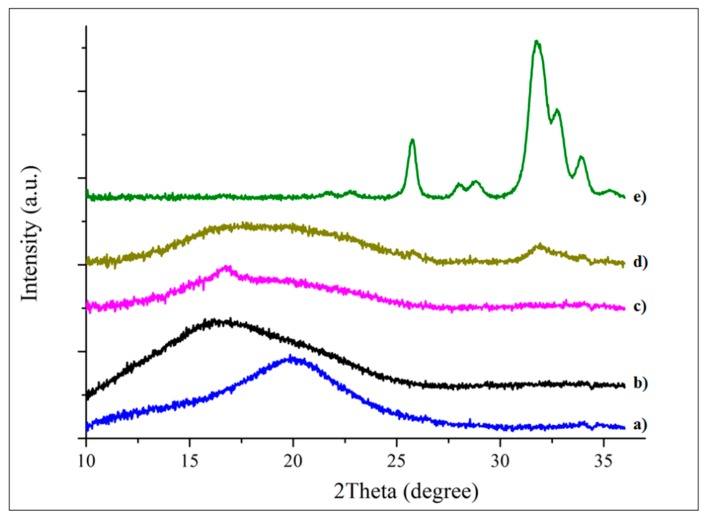
WAXS profiles of the analyzed samples: (**a**) Ge; (**b**) NanoF; (**c**) GeN; (**d**) GeNHAp; (**e**) HAp_powder.

The WAXS diffraction spectra of Ge, GeN, and GeNHAp in comparison to the Ge_powder, NanoF, and HAp_powder are presented in Figure 5. Ge_powder and NanoF indicated a broad amorphous peak at 20 degrees and 16 degrees, respectively. A number of peaks related to HAp crystals were observed; the main diffraction peaks of the HAp appearing at 26.4 degrees, 29.35 degrees, 32 degrees, 33.3 degrees, and 34.2 degree are associated with crystallographic planes such as 002, 102, 112, 300, 202, respectively [38]. Ge indicated a broad halo characteristic for amorphous gelatin [39]. Peaks related to single components appeared on the diffraction spectra of the GeN and GeNHAp specimens. Additionally, the GeN sample indicated a crystal/semi-crystal peak of about 16 degrees, which was not observed in the pure PLGA. The GeNHAp specimen indicated the same peaks as GeN, and, additionally, all the above-mentioned peaks were characteristic for HAp_powder.

**Figure 6:**
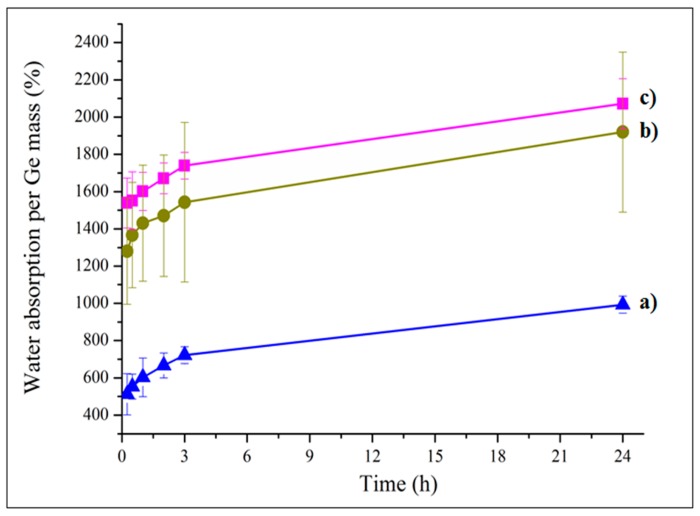
Water absorption of analyzed 3D hybrid scaffolds (**b**) GeNHAp and (**c**) GeN in comparison to the freeze-dried gelatin sample (**a**) Ge.

Water absorption of the analyzed samples GeN, and GeNHAp in comparison to Ge was analyzed for 24 h (Figure 6). PLGA fibers are highly hydrophobic, so water absorption was calculated on a mass of gelatin (Ge) or on a mass of gelatin and HAp (GeNHAp) in the sample bulk. 3D scaffolds with nanofibers indicated a higher water absorption than a pure Ge scaffold. Despite the hydrophilic character of HAp, it contributed to a lower water uptake in comparison to GeN.

**Figure 7:**
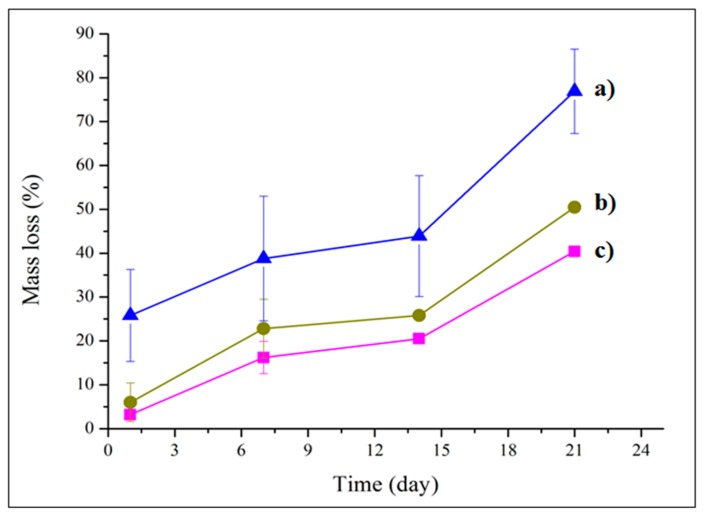
Mass loss of prepared 3D hybrid scaffolds (**c**) GeN and (**b**) GeNHAp in comparison to freeze-dried gelatin sample (**a**) Ge.

The mass loss of the prepared scaffolds GeN and GeNHAp in comparison to Ge was analyzed during 21 days of in vitro experiments (Figure 7). After one day, the GeN and GeNHAp samples indicated a weight loss of approximately 5%, which was three times less than that in the case of Ge. During day 21 of the experiment, 3D hybrid scaffolds (GeN and GeNHAp) indicated a nearly two-fold lower weight loss rate than Ge.

**Figure 8:**
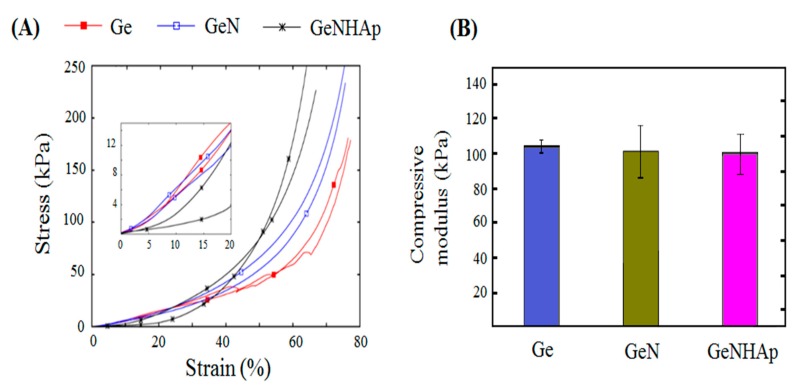
Stress–strain relationship of the 3D hybrid scaffolds (GeN, GeNHAp) determined with DMA: (**A**) compression stress–strain response of the analyzed 3D scaffolds; (**B**) compressive moduli of analyzed scaffolds.

The mechanical properties of the investigated scaffolds were identified. Figure 8A presents the stress–strain curves obtained in a monotonic compressive test. It may be easily seen that all the scaffolds exhibited a non-linear trend of the stress–strain curves. It is hard to distinguish the exact yielding points of the analyzed stress–strain curves. The measured stiffness of all scaffolds was relatively low in the early stage of the compression and increased rapidly in the second stage of the test, which is a characteristic feature of all native extracellular matrices (ECMs) [40]. The compressive moduli identified in the investigated kinds of scaffolds were comparable (Figure 8B). The ultimate compressive stress (UCS) of the Ge samples was determined as 40 kPa. The UCSs of the remaining investigated kinds of samples were not determined due to the fact that the instrument specific force limit was reached before the sample failure.

**Figure 9:**
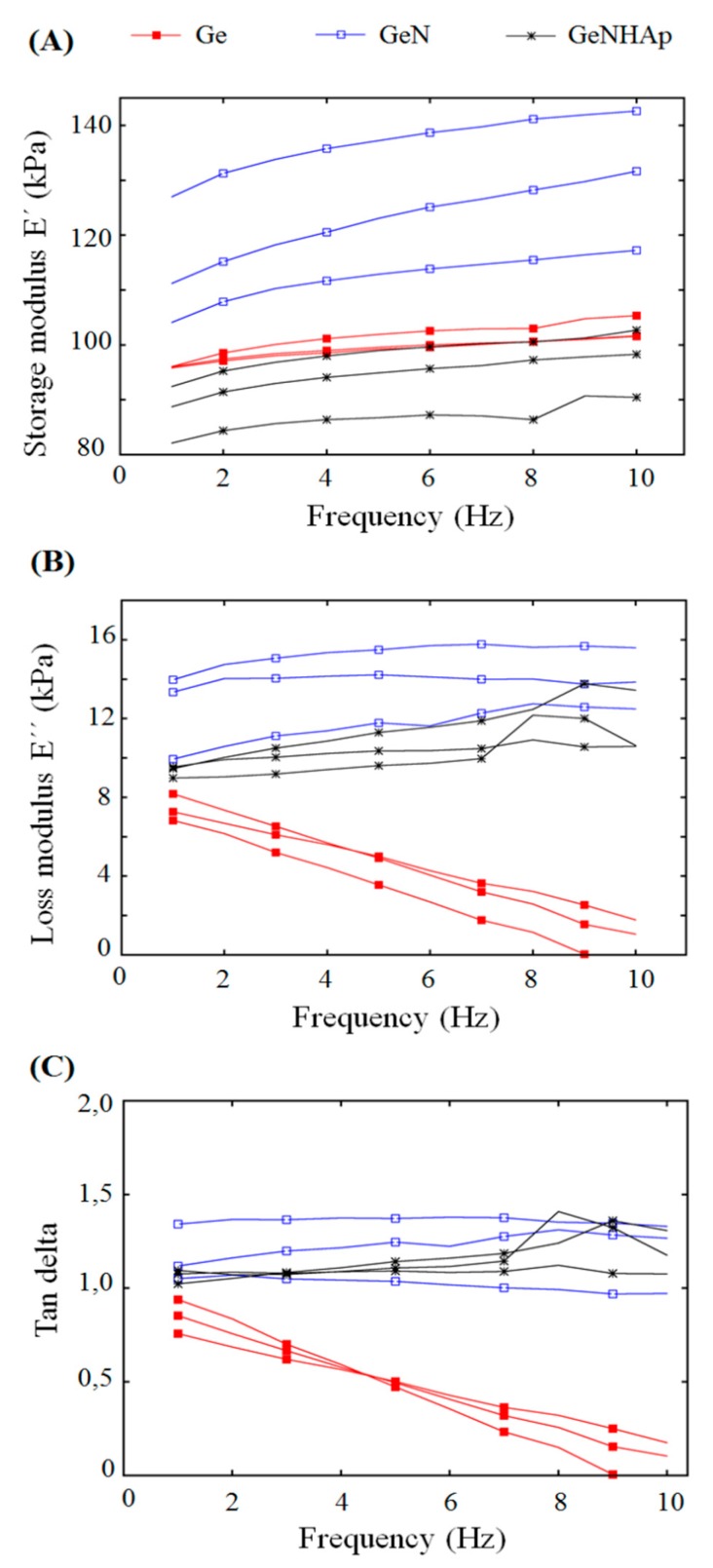
Viscoelastic properties of the 3D hybrid scaffolds (GeN, GeNHAp) and Ge freeze-dried samples determined with DMA. (**A**) Storage modulus as a function of frequency; (**B**) loss modulus as a function of frequency; (**C**) tangent of phase lag as a function of frequency.

To investigate the viscoelastic properties of the scaffolds, dynamic compression tests were performed. All types of the analyzed scaffolds showed a mostly flat response in the storage and the loss modulus in frequency function, with the exception of the Ge samples (Figure 9A,B). The loss modulus of the mentioned groups decreased significantly with frequency. It can be seen that independently of the samples, type *E*’ was much greater than *E*’’, indicating the predomination of elastic material behavior over that of viscous. The identified storage modulus was more differentiated between the groups of samples considered than the compressive moduli obtained in a quasi-static mechanical test. Figure 9A also shows that the GeN scaffolds are characterized by the greatest stiffness expressed in terms of the storage modulus *E*’.

**Figure 10:**
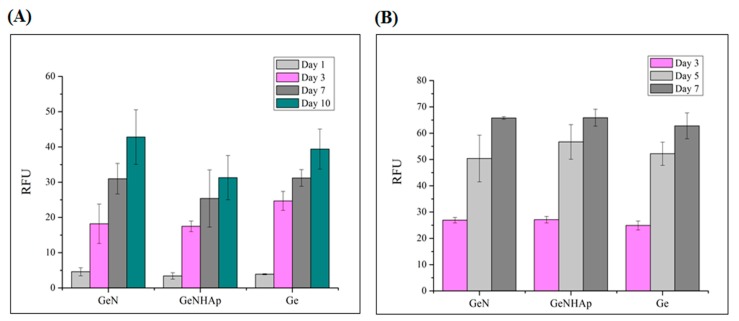
Cytotoxicity analysis of the 3D hybrid scaffolds (GeN, GeNHAp) and the Ge freeze-dried control sample using a Presto Blue assay in direct contact mode for two cell types. (**A**) Mouse fibroblasts; (**B**) Human osteoblasts.

**Figure 11:**
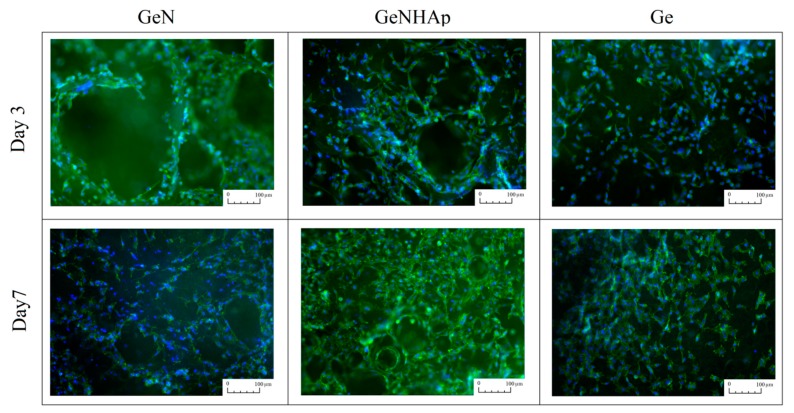
Fluorescent microscope images presenting fibroblasts spreading on the 3D scaffolds (Ge, GeN, and GeNHAp). Observations were performed after three and seven days of cell culture. The actine skeleton was stained green, while the nucleuses were stained blue.

**Figure 12:**
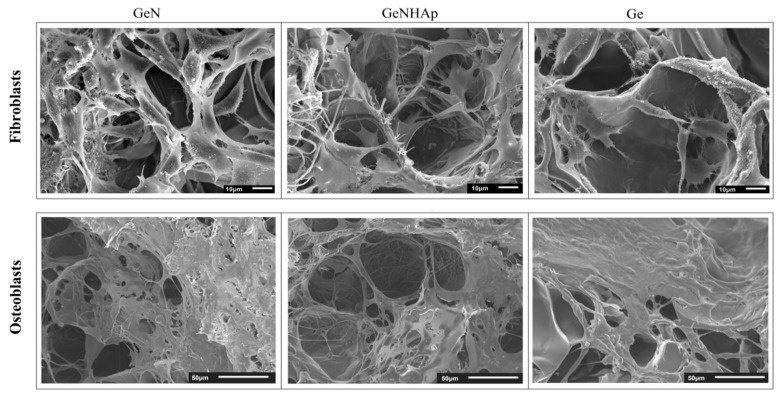
SEM images of cellular morphology after seven days culture on the 3D scaffolds (Ge, GeN, and GeNHAp).

Figure 9 illustrates the cellular viability obtained for mouse fibroblasts (L929) and human osteoblasts (MG63) cultured on 3D scaffolds (Ge, GeN, GeNHAp) analyzed up to 10 days of the experiment (Figure 10). Cellular morphology was illustrated using FM and SEM microscopy and is presented in Figure 11 and Figure 12. All cells were spread on the scaffold surface and migrated inside the scaffold. Cellular lamelliopodium with filopodia were entwined on the scaffold walls. A Presto Blue assay was performed on the samples in contact mode, confirming the nontoxic character of the samples (Figure 10).

## 4. Discussion

The hybrid scaffolds, which are a combination of gelatin, PLGA fibers, and PLGA fibers covered with HAp, were prepared successfully. All scaffolds indicated a high and open porosity, which contributed to the high surface area (Figure 2A). As shown in the SEM images, the PLGA fibers were embedded in the gelatin pore walls, contributing to an increase of surface variability (Figure 3A). The amount of HAp was determined by TGA at c.a 3%, which corresponds to our previous study [33] (Figure 3C).

An EDC/NHS solution in ethanol as a solvent was used to crosslink the scaffolds. EDC is extensively used to trigger the crosslinking of gelatin [41], and NHS-esters were used to yield stable products upon reaction with primary or secondary amines [42]. Lysine has a positively charged ε-amino group with a significantly higher pKa than does the α-amino group [43]. There is an EDC/NHS environmental effect in lowering the pKa of the lysine, which becomes reactive [34]. As was expected, FTIR spectra have proved a new interaction between gelatin chains due to EDC/NHS crosslinking. From the literature, the intensity of the amide I and amide II bands increase with a degree of crosslinking due to the increase of the strength of C=O and N–H vibrations in the new covalent bonds [44,45]. Additionally, under crosslinking conditions, the transformation of –NH_2_ into N–H groups appears and the intensity of the amide II band may decrease since the intensity of the –NH_2_ band is stronger than N–H [46]. However, such changes were not observed (Figure 4). It might be explained by the fact that some of the non-crosslinked peptide elements were washed away [47], there was a lower number of C=O vibrations, and thus a smaller amide I peak area was detected (Figure 4).

Several authors noted that the chemical modification of Ge molecules by a crosslinking agent affected the lattice interaction between organic molecules and apatite interfaces. The FTIR spectral change of –COO– wagging bands at c.a 1400 cm^−1^ showed modification of the e-amine group in the Ge molecule due to the crosslinking with HAp, as observed by Chang et al. [48]. The –COO groups in gelatin make covalent bonds with Ca^2+^ ions in the interfacial surface of HAp [49,50,51,52]. A shift of wavelengths corresponding to amide I and amide II was noticed, and is illustrated in Figure 4. Stanishevsky et al. suggested that those amide I and amide II shifts to higher wavelengths might be associated not only with gelatin crosslinking, but also with the chemical interaction between the apatite nanoparticles and peptides [53].

Additionally, signals from the interaction between gelatin and PLGA fibers were noticed. The carboxyl groups of the PLGA fibers and the amino groups of gelatin interacted due to the presence of EDC/NHS. The same effect was observed by Sadeghi et al. [54], who grafted collagen on the PLGA surface. Carboxyl groups were activated via the formation of the highly reactive O-acylisocarbamide [54]. This agent interacted with the amino groups of collagen nucleophiles and formed stable amide bonds [55]. Change of peak position related to the carbonyl group in the presence of gelatin was detected by FTIR (GeN, GeNHAp) (Figure 4).

According to the theory of equilibrium swelling, the swelling ratio of the gels is related to their effective crosslinking density [56]. Water absorption analysis is generally accepted as a suitable method to estimate the crosslinking density of a hydrogel. The increase in water uptake is related with pH osmotic pressure increase [57,58]. Water uptake of GeN and GeNHAp samples was increased in comparison to Ge (Figure 6). The gelatin crosslinking degree increased due to gelatin crosslinking with EDC/NHS, interactions of gelatin with PLGA carboxyl groups, and Ca^2+^ presence in GeNHAp. Metal ions contributed to the decrease in the gelatin–gelatin crosslinking degree. A few reports have undermined the benefits of divalent metal ion additives [59] where those authors noticed that free carboxylic acid groups in polypeptide molecules improved the crosslinking density between gelatin. In the case of the 3D hybrid scaffolds presented in this article, this effect was observed as a lower water uptake of the GeNHAp sample in comparison to GeN, most likely blocking gelatin crosslinking by Ca^2+^ ions (Figure 6). On the other hand, PLGA and hydroxyapatite change the pH of the gelatin environment under swelling conditions. PLGA indicates a slightly acetic pH as hydroxyapatite has the ability to increase pH under in vitro conditions [60]. Under acidic pH conditions, mostly carboxylate anions are protonated [61]. As a result, anion–anion repulsive forces are eliminated and consequently, water uptake decreases. With an increase of pH up to 7, some of the carboxylate groups are ionized and the electrostatic repulsion between COO– groups, causing an enhancement of the swelling capacity. Below pH 7, a charge screening effect of the counter cations leads to swelling limitation [61]. This effect was analyzed during the hydrogel development [62,63]. In our case, an increase in the crosslinking degree of gelatin with PLGA and changes in pH due to the presence of PLA/HAp were observed. We suppose that an increase in the surface area due to the fiber additive was also important for the swelling degree of the analyzed 3D hybrid scaffolds (Figure 2A and Figure 6).

In general, mass loss of gelatin is tailored by the temperature, pH, and gelatin properties, but mainly isoelectric point and molecular weight (Mw). Gelatin is a protein with an isoelectric point between 5 and 9 [64]. In the manufacturing process, some glutamine and asparagine are converted to acidic forms. This process leads to an increase in the number of carboxyl groups, thus lowering the isoelectric point [65]. The difference in the solubility of different gelatins might result from the differences in M_W_ and the content of polar and non-polar groups in amino acids [66]. We proved a higher crosslinking degree of GeN and GeNHAp than Ge (Figure 4, Figure 7). Additionally, changes of pH under in vitro conditions in the presence of PLGA fibers were expected. As a consequence, the mass loss of samples with fibers (GeN and GeNHAp) was much lower than Ge (Figure 7).

It might be expected that the bincorporation of hydrophobic PLGA nanofibers could result in significantly decreased scaffold water absorbance. However, one can see just the opposite effect. A reasonable explanation of the observed facts could be that it is attributed to the potential effect of the presence of hydrophobic PLGA nanofibers in the freeze-drying process. Banerjee et al. [67] proved that the presence of hydrophobic PLGA microspheres in a gelatin matrix could alter the size and distribution of ice-crystals formed at the time of freezing. As a consequence, the porosity as well as the microarchitecture of the gelatin scaffold matrix could be changed significantly. The above-mentioned hypothesis might suggest that incorporation of PLGA nanofibers could increase the scaffold porosity so that it allows it to absorb more water. Confirmation of this hypothesis could lead to the fact that incorporation of PLGA nanofibers coated by HAp, which are more wettable than uncoated PLGA nanofibers, leads to much less water absorbance than in the case of a gelatin matrix filled with uncoated PLGA nanofibers. Similarly, as in [67], the mechanical properties of the gelatin hybrid scaffold could be corelated to its porosity/water absorbance. An example of such a relation is present in Figure 5 and Figure 9A, which present the water absorbance and storage modulus, respectively.

The in vitro studies, conducted in direct contact with mouse fibroblasts recommended in biocompatibility assessment (ISO 10993-5), confirmed the nontoxic character of the 3D hybrid scaffolds (Figure 10, Figure 11 and Figure 12). The viability test confirmed that both cell types, fibroblasts, and osteoblasts proliferate constantly during the experiment time on all 3D scaffolds (Figure 10A,B). Moreover, at each time point, the cell proliferation on these samples was comparable. The SEM images and fluorescent microscopy confirmed that cells cultured the whole scaffold bulk (Figure 11 and Figure 12). Additionally, nice spreading, proliferation, and migration of human osteoblasts proved their potential for application in bone regeneration. Due to the increase of nonwoven porosity, cellular migration was enhanced. The chosen combination of peptide, polyester fibers, and HAp as well as a solid 3D structure of the scaffold might be a good alternative for currently applied scaffolds in tooth implant fixation [68].

## 5. Conclusions

We demonstrated that the development of hybrid scaffolds is more complex from a structural point of view than that of a morphological. The presence of polyesters and Ca^2+^ ions changes the natural neighborhood of gelatin molecules, pH, polarity of surrounding groups, water amount, etc. As an amphoteric polyelectrolyte, gelatin is interfacially active and its activity depends on a balance of positive and negative groups. Additionally, the crosslinking degree resulted in slower mass loss in in vitro studies, which is beneficial from the viewpoint of practical use of the scaffold.

Due to the use of both materials types, the biodegradation time was differentiated. The gelatin exhibited significant mass loss within a few weeks, while due to the fibers’ presence in the 3D scaffold, additional hydrogen bonds occurred, thus extending the scaffold stability. Moreover, the PLGA fibers will provide support for cellular growth much longer than gelatin, up to a few months [69]. HAp ions did not have such a significant influence on scaffold stability. Hybrid scaffolds not only combine the advantages of all components, but also exhibit new features, for example, biodegradation time and mechanical properties, which arise from new interactions occurring between the scaffold components. One important impact on the scale of new interactions is also the selected scaffold preparation methodology and processing conditions. The 3D hybrid scaffolds presented are a promising alternative for materials used in medical applications such as bone regeneration.
